# Supplementary figures and images for: The rapid developmental rise of somatic inhibition disengages hippocampal dynamics from self-motion
Source: eLife. 2022 Jul 20;11:e78116. doi: 10.7554/eLife.78116 (PMC9363116; doi:10.7554/eLife.78116)

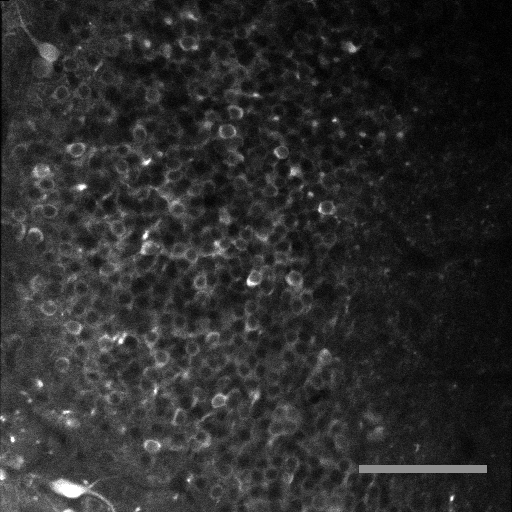

Supplement: Figure 1—source data 1. — (A) Field of views and the Calcium Imaging Complete Automated Data Analysis (CICADA) configuration files necessary to plot the contours map and raster plots used for the illustration. (B) Numerical data used to plot the evolution of the transient in synchronous calcium event (SCE) and the CICADA configuration file necessary to reproduce the analysis. (C) Numerical data used to plot the evolution of the transient per minute and the CICADA configuration file necessary to reproduce the analysis. [file elife-78116-fig1-data1.zip › Figure1 - Source Data 1/1A/p10_hd.tif]

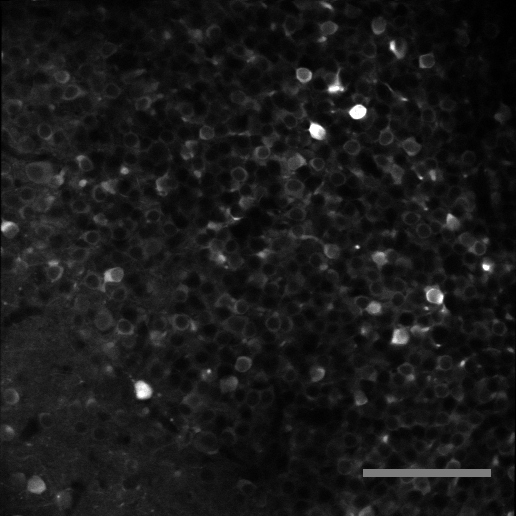

Supplement: Figure 1—source data 1. — (A) Field of views and the Calcium Imaging Complete Automated Data Analysis (CICADA) configuration files necessary to plot the contours map and raster plots used for the illustration. (B) Numerical data used to plot the evolution of the transient in synchronous calcium event (SCE) and the CICADA configuration file necessary to reproduce the analysis. (C) Numerical data used to plot the evolution of the transient per minute and the CICADA configuration file necessary to reproduce the analysis. [file elife-78116-fig1-data1.zip › Figure1 - Source Data 1/1A/p12_hd.tif]

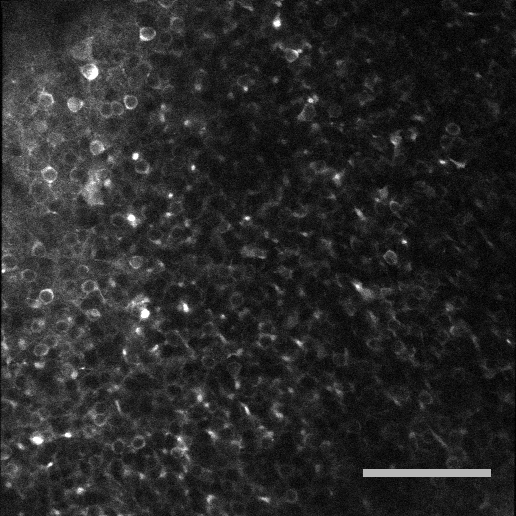

Supplement: Figure 1—source data 1. — (A) Field of views and the Calcium Imaging Complete Automated Data Analysis (CICADA) configuration files necessary to plot the contours map and raster plots used for the illustration. (B) Numerical data used to plot the evolution of the transient in synchronous calcium event (SCE) and the CICADA configuration file necessary to reproduce the analysis. (C) Numerical data used to plot the evolution of the transient per minute and the CICADA configuration file necessary to reproduce the analysis. [file elife-78116-fig1-data1.zip › Figure1 - Source Data 1/1A/p5_hd.tif]

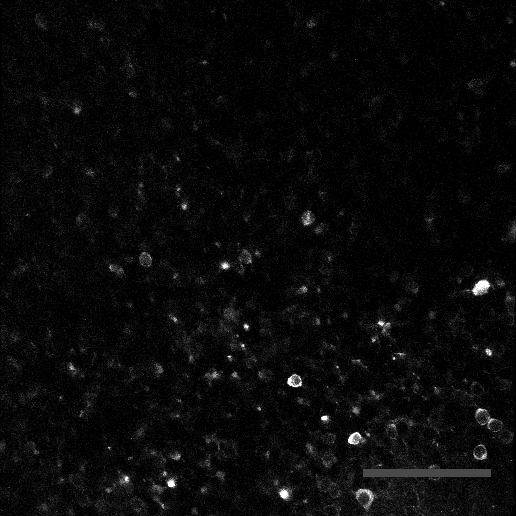

Supplement: Figure 1—source data 1. — (A) Field of views and the Calcium Imaging Complete Automated Data Analysis (CICADA) configuration files necessary to plot the contours map and raster plots used for the illustration. (B) Numerical data used to plot the evolution of the transient in synchronous calcium event (SCE) and the CICADA configuration file necessary to reproduce the analysis. (C) Numerical data used to plot the evolution of the transient per minute and the CICADA configuration file necessary to reproduce the analysis. [file elife-78116-fig1-data1.zip › Figure1 - Source Data 1/1A/p7_hd.tif]

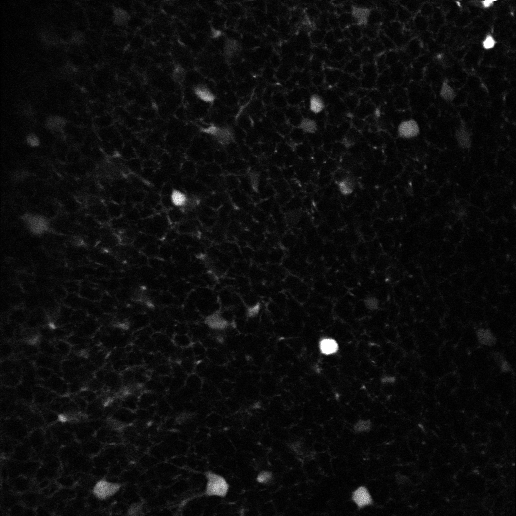

Supplement: Figure 3—source data 1. — ‘example_FoVs’: images used for illustration in Figure 3. ‘example_raster_plots’: two Calcium Imaging Complete Automated Data Analysis (CICADA) configuration files necessary to reproduce the raster plots used for illustration in Figure 3. ‘psths’: numerical data used to plot Figure 3 peri-movement time histograms (PMTHs) and the CICADA configuration file necessary to reproduce the analysis. [file elife-78116-fig3-data1.zip › Figure3 - Source Data 1/example_FoVs/example_P12_composite.tif]

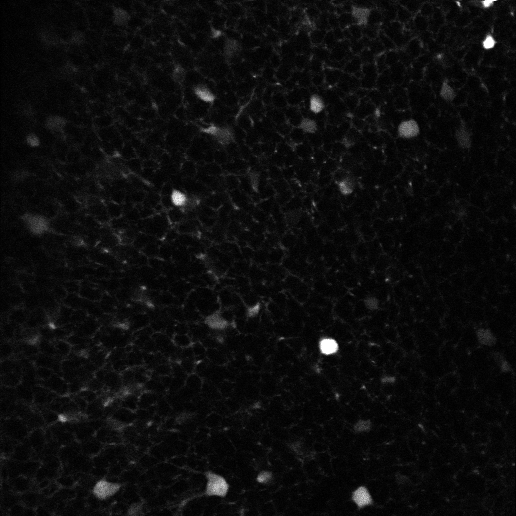

Supplement: Figure 3—source data 1. — ‘example_FoVs’: images used for illustration in Figure 3. ‘example_raster_plots’: two Calcium Imaging Complete Automated Data Analysis (CICADA) configuration files necessary to reproduce the raster plots used for illustration in Figure 3. ‘psths’: numerical data used to plot Figure 3 peri-movement time histograms (PMTHs) and the CICADA configuration file necessary to reproduce the analysis. [file elife-78116-fig3-data1.zip › Figure3 - Source Data 1/example_FoVs/example_P12_tdtomato.tif]

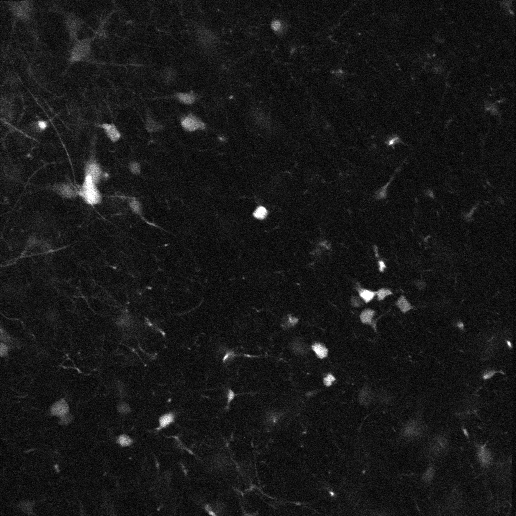

Supplement: Figure 3—source data 1. — ‘example_FoVs’: images used for illustration in Figure 3. ‘example_raster_plots’: two Calcium Imaging Complete Automated Data Analysis (CICADA) configuration files necessary to reproduce the raster plots used for illustration in Figure 3. ‘psths’: numerical data used to plot Figure 3 peri-movement time histograms (PMTHs) and the CICADA configuration file necessary to reproduce the analysis. [file elife-78116-fig3-data1.zip › Figure3 - Source Data 1/example_FoVs/example_P5_composite.tif]

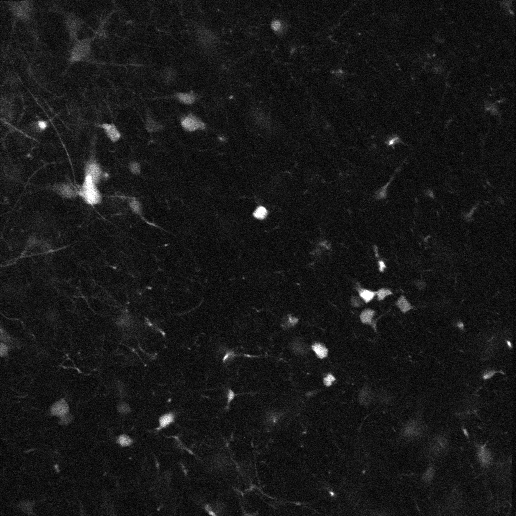

Supplement: Figure 3—source data 1. — ‘example_FoVs’: images used for illustration in Figure 3. ‘example_raster_plots’: two Calcium Imaging Complete Automated Data Analysis (CICADA) configuration files necessary to reproduce the raster plots used for illustration in Figure 3. ‘psths’: numerical data used to plot Figure 3 peri-movement time histograms (PMTHs) and the CICADA configuration file necessary to reproduce the analysis. [file elife-78116-fig3-data1.zip › Figure3 - Source Data 1/example_FoVs/example_P5_tdtomato.tif]
