# Supplementary material for: The rapid developmental rise of somatic inhibition disengages hippocampal dynamics from self-motion
Source: eLife. 2022 Jul 20;11:e78116. doi: 10.7554/eLife.78116 (PMC9363116; doi:10.7554/eLife.78116)
Supplement: Figure 5—source data 1. [file elife-78116-fig5-data1.zip › Figure_5_Source_data_1.pdf]

Parameter	Symbol	Value
**Rate model**		
Excitatory time constant	τ_E	0.1 s
Inhibitory time constant	τ_I	0.8 s
Connectivity matrix		
	J_{EE}	$5.0\,s^{-1}$
	J_{IE}	$2.0\,s^{-1}$
	J_{II}	$-0.8\,s^{-1}$
	J_{EI}^{weak}	$-0.0\,s^{-1}$
	J_{EI}^{strong}	$-1.6\,s^{-1}$
Noise amplitude	η	$0.25\,s^{-1/2}$
External input		
	H_E	$3.5\,s^{-2}$
	H_I	$0.4\,s^{-2}$
External drive increase	α	3.0
Twitch amplitude		
	J_E	$50.0\,s^{-2}$
	J_I	$100.0\,s^{-2}$
**LIF model**		
Exc. synapse time constant	τ_E	100.0 ms
Inh. synapse time constant	τ_I	800.0 ms
Membrane time constant	τ_m	20.0 ms
Threshold potential	V_{thr}	20.0 mV
Reset potential	V_{reset}	0.0 mV
Connectivity		
Nb. of excitatory neurons	N_E	1024
Nb. of inhibitory neurons	N_I	256
Connections from exc. neurons	K_E	64
Connections from inh. neurons	K_I	32
Connectivity matrix		
	J_{EE}	0.025 mV
	J_{IE}	0.1 mV
	J_{II}	−0.0 mV
	J_{EI}^{weak}	−0.0005 mV
	J_{EI}^{strong}	−0.005 mV
Noise amplitude	η	$1.8\,ms^{-1/2}$
External input (weak inhibition)		
	H_E	$-0.07\,mV\,ms^{-1}$
	H_I	$0.015\,mV\,ms^{-1}$
External input (strong inhibition)		
	H_E	$0.04\,mV\,ms^{-1}$
	H_I	$0.06\,mV\,ms^{-1}$
Twitch amplitude		
	J_E	$0.2\,mV\,ms^{-1}$
	J_I	$0.2\,mV\,ms^{-1}$
Twitch duration (lognormal)		
Mean parameter	μ	−0.2
Std parameter	σ	0.8
