# Supplementary material for: The rapid developmental rise of somatic inhibition disengages hippocampal dynamics from self-motion
Source: eLife. 2022 Jul 20;11:e78116. doi: 10.7554/eLife.78116 (PMC9363116; doi:10.7554/eLife.78116)
Supplement: Supplementary file 1. — * indicates mouse pups that are used for illustration. Y, included in the panel; N, not included. [file elife-78116-supp1.docx]

|  |  |  |  | **Main Figures** | | | | | | | | | | |  |
| --- | --- | --- | --- | --- | --- | --- | --- | --- | --- | --- | --- | --- | --- | --- | --- |
| **Age** | **SubjectID** | **SessionID** | **N_Cells** | **1A** | **1B** | **1C** | **2A** | **2B** | **2C** | **2D** | **3A** | **3B** | **4C** | **4D** | **5C** |
| 5 | 190320_190325 | 190325_a000 | 633 | N | Y | Y | Y | Y | Y | Y | Y | N | N | N | Y |
|  |  | 190325_a001 | 919 | N | Y | Y | Y | Y | Y | Y | Y | N | N | N | Y |
| 5 | 191205_191210_0 | 191210_a000 | 316 | Y | Y | Y | Y | Y | Y | Y | Y* | N | N | N | Y |
|  |  | 191210_a001 | 128 | N | Y | Y | Y | Y | Y | Y | Y | N | N | N | Y |
| 5 | 191205_191210_1 | 191210_a000 | 512 | N | Y | Y | Y | Y | Y | Y | Y | N | N | N | Y |
|  |  | 191210_a001 | 117 | N | Y | Y | Y | Y | Y | Y | Y | N | N | N | Y |
| 5 | 200306_200311 | 200311_a000 | 388 | N | Y | Y | Y | Y | Y | Y | Y | N | N | N | Y |
|  |  | 200311_a001 | 272 | N | Y | Y | Y | Y | Y | Y | Y | N | N | N | N |
| 6 | 180201_180207 | 180207_a001 | 566 | N | Y | Y | Y | Y | Y | Y | Y | N | N | N | Y |
|  |  | 180207_a002 | 405 | N | Y | Y | Y | Y | Y | Y | Y | N | N | N | Y |
| 6 | 190921_190927_1 | 190927_a000 | 240 | N | Y | Y | Y | Y | Y | N | Y | N | N | N | N |
| 6 | 191205_191211_1 | 191211_a000 | 566 | N | Y | Y | Y | Y | Y | Y | Y | N | N | N | Y |
|  |  | 191211_a001 | 395 | N | Y | Y | Y | Y | Y | Y | Y | N | N | N | Y |
| 7 | 180201_180208 | 180208_a000 | 515 | N | Y | Y | Y | Y | Y | Y | Y | N | N | N | Y |
|  |  | 180208_a001 | 846 | N | Y | Y | Y | Y | Y | Y | Y | N | N | N | Y |
|  |  | 180208_a002 | 742 | N | Y | Y | Y | Y | Y | Y | Y | N | N | N | Y |
|  |  | 180208_a003 | 622 | N | Y | Y | Y | Y | Y | Y | Y | N | N | N | Y |
| 7 | 190226_190305 | 190305_a000 | 410 | N | Y | Y | Y | Y | Y | Y | Y | N | N | N | Y |
| 7 | 190320_190327 | 190327_a000 | 824 | N | Y | Y | Y | Y | Y | Y | Y | N | N | N | Y |
|  |  | 190327_a001 | 678 | N | Y | Y | Y | Y | Y | Y | Y | N | N | N | Y |
|  |  | 190327_a002 | 655 | N | Y | Y | Y | Y | Y | Y | Y | N | N | N | Y |
| 7 | 200103_200110_0 | 200110_a000 | 453 | N | Y | Y | Y | Y | Y | Y | Y | N | N | N | Y |
|  |  | 200110_a001 | 486 | Y | Y | Y | Y | Y | Y | Y | Y | N | N | N | Y |
| 7 | 200206_200213 | 200213_a000 | 611 | N | Y | Y | Y | Y | Y | Y | Y | N | N | N | Y |
|  |  | 200213_a001 | 322 | N | Y | Y | Y | Y | Y | Y | Y | N | N | N | N |
| 8 | 181009_181017 | 181017_a000 | 627 | N | Y | Y | Y | Y | Y | Y | Y | N | N | N | Y |
|  |  | 181017_a001 | 588 | N | Y | Y | Y | Y | Y | Y | Y | N | N | N | Y |
| 8 | 181016_181024 | 181024_a005 | 565 | N | Y | Y | Y | Y | Y | Y | Y | N | N | N | Y |
| 8 | 190921_190929_1 | 190929_a000 | 698 | N | Y | Y | Y | Y | Y | Y | Y | N | N | N | Y |
| 8 | 191205_191213 | 191213_a000 | 712 | N | Y | Y | Y | Y | Y | Y | Y | N | N | N | N |
|  |  | 191213_a001 | 297 | N | Y | Y | Y | Y | Y | Y | Y | N | N | N | N |
| 8 | 191212_191220 | 191220_a001 | 537 | N | Y | Y | Y | Y | Y | Y | Y | N | N | N | Y |
|  |  | 191220_a003 | 620 | N | Y | Y | Y | Y | Y | Y | Y | N | N | N | Y |
| 9 | 171211_171220 | 171220_a001 | 372 | N | Y | Y | Y | Y | Y | Y | N | N | N | N | Y |
| 9 | 190211_190220_0 | 190220_a000 | 378 | N | Y | Y | Y | Y | Y | Y | N | N | N | N | Y |
|  |  | 190220_a001 | 942 | N | Y | Y | Y | Y | Y | Y | N | N | N | N | Y |
|  |  | 190220_a002 | 813 | N | Y | Y | Y | Y | Y | Y | N | N | N | N | Y |
|  |  | 190220_a003 | 797 | N | Y | Y | Y | Y | Y | Y | N | N | N | N | Y |
| 9 | 190305_190314 | 190314_a000 | 443 | N | Y | Y | Y | Y | Y | Y | N | N | N | N | Y |
|  |  | 190314_a001 | 500 | N | Y | Y | Y | Y | Y | Y | N | N | N | N | Y |
| 9 | 190313_190322 | 190322_a000 | 844 | N | Y | Y | Y | Y | Y | Y | N | N | N | N | Y |
|  |  | 190322_a001 | 696 | N | Y | Y | Y | Y | Y | Y | N | N | N | N | Y |
| 9 | 190921_190930 | 190930_a001 | 1050 | N | Y | Y | Y | Y | Y | Y | N | N | N | N | N |
|  |  | 190930_a003 | 849 | N | Y | Y | Y | Y | Y | Y | N | N | N | N | N |
| 9 | 200108_200117_1 | 200117_a001 | 34 | N | N | N | N | N | N | N | N | N | N | Y | N |
| 9 | 210226_210307_1 | 210307_a000 | 36 | N | N | N | N | N | N | N | N | N | Y | Y | N |
| 10 | 190211_190221_0 | 190221_a003 | 607 | N | Y | Y | N | N | N | N | N | N | N | N | N |
|  |  | 190221_a005 | 928 | N | Y | Y | N | N | N | N | N | N | N | N | N |
| 10 | 190226_190308 | 190308_a000 | 546 | N | Y | Y | N | N | N | N | N | N | N | N | N |
|  |  | 190308_a001 | 596 | N | Y | Y | N | N | N | N | N | N | N | N | N |
| 10 | 190921_191001 | 191001_a000 | 486 | Y | Y | Y | Y | Y | Y | Y | N | Y | N | N | Y |
| 10 | 210226_210308_1 | 210308_a000 | 34 | N | N | N | N | N | N | N | N | N | N | Y | N |
| 11 | 190211_190222 | 190222_a000 | 606 | N | Y | Y | N | N | N | N | N | N | N | N | N |
| 11 | 190921_191002 | 191002_a001 | 567 | N | Y | Y | Y | Y | Y | Y | N | Y | N | N | Y |
| 12 | 171029_171110 | 171110_a000 | 404 | N | Y | Y | Y | Y | Y | Y | N | Y | N | N | Y |
|  |  | 171110_a002 | 654 | N | Y | Y | N | N | N | N | N | N | N | N | N |
| 12 | 190828_190909 | 190909_a000 | 396 | N | Y | Y | Y | Y | Y | Y | N | Y | N | N | N |
|  |  | 190909_a001 | 430 | N | Y | Y | Y | Y | Y | Y | N | Y | N | N | N |
| 12 | 190911_190923 | 190923_a001 | 413 | N | Y | Y | Y | Y | Y | Y | N | Y | N | N | Y |
| 12 | 190921_191003 | 191003_a001 | 294 | N | Y | Y | Y | Y | Y | Y | N | Y | N | N | N |
| 12 | 191122_191204 | 191204_a000 | 782 | N | Y | Y | Y | Y | Y | Y | N | Y* | N | N | Y |
|  |  | 191204_a001 | 650 | Y | Y | Y | Y | Y | Y | Y | N | Y | N | N | Y |
