# Supplementary material for: The rapid developmental rise of somatic inhibition disengages hippocampal dynamics from self-motion
Source: eLife. 2022 Jul 20;11:e78116. doi: 10.7554/eLife.78116 (PMC9363116; doi:10.7554/eLife.78116)
Supplement: Supplementary file 2. — * shows mouse pups that were used for illustration. Y, included in the panel; N, not included. [file elife-78116-supp2.docx]

|  |  |  | | | | |  |  |  |  |  |  |  |  |  |  |
| --- | --- | --- | --- | --- | --- | --- | --- | --- | --- | --- | --- | --- | --- | --- | --- | --- |
| **SubID** | **SesID** | **1S**  **1A** | **1S**  **1B** | **1S**  **1D** | **1S**  **1E** | **2S**  **1A, B** | | **2S**  **1C** | **2S**  **1E** | **3S**  **1A** | **3S**  **1B** | **3S**  **1D** | **3S**  **1E** | **3S**  **2A** | **3S**  **2B** | **4S**  **1A** |
| 190320_  190325 | 190325_  a000 | Y | N | N | N | Y | | N | Y | N | N | Y | N | Y | N | N |
|  | 190325_  a001 | Y | N | N | N | Y | | N | Y | N | N | Y | N | Y | N | N |
| 191205_  191210_0 | 191210_  a000 | Y | N | Y | N | Y | | N | Y | Y | N | Y | Y | Y | N | N |
|  | 191210_  a001 | Y | N | N | Y | Y | | N | Y | Y | N | Y | Y | Y | N | N |
| 191205_  191210_1 | 191210_  a000 | Y | N | N | N | Y | | N | Y | Y | N | Y | Y | Y | N | N |
|  | 191210_  a001 | Y | N | N | N | Y | | N | Y | Y | N | Y | Y | Y | N | N |
| 200306_  200311 | 200311_  a000 | Y | Y | N | Y | Y | | Y | Y | N | N | Y | Y | Y | N | N |
|  | 200311_  a001 | Y | Y | N | Y | Y | | Y | Y | Y | N | Y | Y | Y | N | Y |
| 180201_  180207 | 180207_  a001 | Y | N | N | N | Y | | N | Y | N | N | Y | N | Y | N | N |
|  | 180207_  a002 | Y | N | N | N | Y | | N | Y | N | N | Y | N | Y | N | N |
| 190921_  190927_1 | 190927_  a000 | Y | N | N | Y | Y | | N | N | N | N | Y | Y | Y | N | N |
| 191205_  191211_1 | 191211_  a000 | Y | N | N | N | Y | | N | Y | N | N | Y | Y | Y | N | N |
|  | 191211_  a001 | Y | N | N | N | Y | | N | Y | Y | N | Y | N | Y | N | N |
| 180201_  180208 | 180208_  a000 | Y | N | N | N | Y | | N | Y | N | N | Y | N | Y | N | N |
|  | 180208_  a001 | Y | N | N | N | Y | | N | Y | N | N | Y | N | Y | N | N |
|  | 180208_  a002 | Y | N | N | N | Y | | N | Y | N | N | Y | N | Y | N | N |
|  | 180208_  a003 | Y | N | N | N | Y | | N | Y | N | N | Y | N | Y | N | N |
| 190226_  190305 | 190305_  a000 | Y | N | N | N | Y | | N | Y | N | N | Y | N | Y | N | N |
| 190320_  190327 | 190327_  a000 | Y | N | N | N | Y | | N | Y | N | N | Y | N | Y | N | N |
|  | 190327_  a001 | Y | N | N | N | Y | | N | Y | N | N | Y | N | Y | N | N |
|  | 190327_  a002 | Y | N | N | N | Y | | N | Y | N | N | Y | N | Y | N | N |
| 200103_  200110_0 | 200110_  a000 | Y | N | N | Y | Y | | N | Y | Y | N | Y | Y | Y | N | N |
|  | 200110_  a001 | Y | N | Y | N | Y | | N | Y | Y | N | Y | Y | Y | N | N |
| 200206_  200213 | 200213_  a000 | Y | N | N | N | Y | | N | Y | N | N | Y | Y | Y | N | N |
|  | 200213_  a001 | Y | N | N | N | Y | | N | Y | N | Y | Y | Y | Y | N | N |
| 181009_  181017 | 181017_  a000 | Y | N | N | N | Y | | N | Y | N | N | Y | N | Y | N | N |
|  | 181017_  a001 | Y | N | N | N | Y | | N | Y | N | N | Y | N | Y | N | N |
| 181016_  181024 | 181024_  a005 | Y | N | N | N | Y | | N | Y | N | N | Y | N | Y | N | N |
| 190921_  190929_1 | 190929_  a000 | Y | N | N | Y | Y | | N | Y | Y | N | Y | Y | Y | N | N |
| 191205_  191213 | 191213_  a000 | Y | N | N | N | Y | | N | Y | N | N | Y | Y | Y | N | N |
|  | 191213_  a001 | Y | N | N | N | Y | | N | Y | N | N | Y | Y | Y | N | N |
| 191212_  191220 | 191220_  a001 | Y | N | N | N | Y | | N | Y | Y | N | Y | Y | Y | N | N |
|  | 191220_  a003 | Y | N | N | N | Y | | N | Y | Y | N | Y | Y | Y | N | N |
| 171211_  171220 | 171220_  a001 | Y | N | N | N | Y | | N | Y | N | N | Y | N | N | N | N |
| 190211_  190220_0 | 190220_  a000 | Y | N | N | N | Y | | N | Y | N | N | Y | N | N | N | N |
|  | 190220_  a001 | Y | N | N | N | Y | | N | Y | N | N | Y | N | N | N | N |
|  | 190220_  a002 | Y | N | N | N | Y | | N | Y | N | N | Y | N | N | N | N |
|  | 190220_  a003 | Y | N | N | N | Y | | N | Y | N | N | Y | N | N | N | N |
| 190305_  190314 | 190314_  a000 | Y | N | N | N | Y | | N | Y | N | N | Y | N | N | N | N |
|  | 190314_  a001 | Y | N | N | N | Y | | N | Y | N | N | Y | N | N | N | N |
| 190313_  190322 | 190322_  a000 | Y | N | N | N | Y | | N | Y | N | N | Y | N | N | N | N |
|  | 190322_  a001 | Y | N | N | N | Y | | N | Y | N | N | Y | N | N | N | N |
| 190921_  190930 | 190930_  a001 | Y | N | N | N | Y | | N | Y | N | N | Y | Y | N | N | N |
|  | 190930_  a003 | Y | N | N | N | Y | | N | Y | N | N | Y | Y | N | N | N |
| 200108_  200117_1 | 200117_  a001 | N | N | N | N | N | | N | N | N | N | N | N | N | N | N |
| 210226_  210307_1 | 210307_  a000 | N | N | N | N | N | | N | N | N | N | N | N | N | N | N |
| 190211_  190221_0 | 190221_  a003 | Y | N | N | N | N | | N | N | N | N | Y | N | N | N | N |
|  | 190221_  a005 | Y | N | N | N | N | | N | N | N | N | Y | N | N | N | N |
| 190226_  190308 | 190308_  a000 | Y | N | N | N | N | | N | N | N | N | Y | N | N | N | N |
|  | 190308_  a001 | Y | N | N | N | N | | N | N | N | N | Y | N | N | N | N |
| 190921_  191001 | 191001_  a000 | Y | N | Y | N | Y | | N | Y | Y | N | Y | Y | N | Y | N |
| 210226_  210308_1 | 210308_  a000 | N | N | N | N | N | | N | N | N | N | N | N | N | N | N |
| 190211_  190222 | 190222_  a000 | Y | N | N | N | N | | N | N | N | N | Y | N | N | N | N |
| 190921_  191002 | 191002_  a001 | Y | N | N | N | Y | | N | Y | Y | N | Y | Y | N | Y | N |
| 171029_  171110 | 171110_  a000 | Y | N | N | N | Y | | N | Y | N | Y | Y | N | N | Y | N |
|  | 171110_  a002 | Y | N | N | N | N | | N | N | N | N | Y | N | N | N | N |
| 190828_  190909 | 190909_  a000 | Y | N | N | N | Y | | N | Y | N | N | Y | N | N | Y | N |
|  | 190909_  a001 | Y | N | N | N | Y | | N | Y | N | N | Y | N | N | Y | N |
| 190911_  190923 | 190923_  a001 | Y | N | N | N | Y | | N | Y | N | N | Y | N | N | Y | N |
| 190921_  191003 | 191003_  a001 | Y | N | N | N | Y | | N | Y | N | N | Y | Y | N | Y | N |
| 191122_  191204 | 191204_  a000 | Y | N | N | N | Y | | N | Y | Y | N | Y | Y | N | Y | N |
|  | 191204_  a001 | Y | N | Y | N | Y | | N | Y | Y | N | Y | Y | N | Y | Y |
